# ‘The influence of gestational age and socioeconomic status on neonatal outcomes in late preterm and early term gestation: a population based study’

**DOI:** 10.1186/1471-2393-12-62

**Published:** 2012-06-29

**Authors:** Chelsea A Ruth, Noralou Roos, Elske Hildes-Ripstein, Marni Brownell

**Affiliations:** 1 Section of Neonatology, University of Manitoba, Winnipeg, MB, Canada; 2 Department of Community Health Sciences, University of Manitoba, Winnipeg, MB, Canada; 3 Department of Paediatrics and Child Health, University of Manitoba, Winnipeg, MB, Canada

## Abstract

**Background:**

Infants born late preterm (34 + 0 to 36 + 6 weeks GA (gestational age)) are known to have higher neonatal morbidity than term (37 + 0 to 41 + 6 weeks GA) infants. There is emerging evidence that these risks may not be homogenous within the term cohort and may be higher in early term (37 + 0 to 38 + 6 weeks GA). These risks may also be affected by socioeconomic status, a risk factor for preterm birth.

**Methods:**

A retrospective population based cohort of infants born at 34 to 41 weeks of GA was assembled; individual and area-level income was used to develop three socioeconomic (SES) groups. Neonatal morbidity was grouped into respiratory distress syndrome (RDS), other respiratory disorders, other complications of prematurity, admission to a Level II/III nursery and receipt of phototherapy. Regression models were constructed to examine the relationship of GA and SES to neonatal morbidity while controlling for other perinatal variables.

**Results:**

The cohort contained 25 312 infants of whom 6.1% (n = 1524) were born preterm and 32.4% (n = 8203) were of low SES. Using 39/40 weeks GA as the reference group there was a decrease in neonatal morbidity at each week of gestation. The odds ratios remained significantly higher at 37 weeks for RDS or other respiratory disorders, and at 38 weeks for all other outcomes. SES had an independent effect, increasing morbidity with odds ratios ranging from 1.2–1.5 for all outcomes except for the RDS group, where it was not significant.

**Conclusions:**

The risks of morbidity fell throughout late preterm and early term gestation for both respiratory and non-respiratory morbidity. Low SES was associated with an independent increased risk. Recognition that the morbidities associated with prematurity continue into early term gestation and are further compounded by SES is important to develop strategies for improving care of early term infants, avoiding iatrogenic complications and prioritizing public health interventions.

## Background

Risks of neonatal morbidity related to maturity fall with each week of gestation throughout the late preterm period [1] but little is known about how this gradient acts past 36 weeks. A small number of studies have demonstrated persistent risks in early term gestation (defined as 37 to 38 completed weeks) casting doubt on the practice of considering all infants born at 37 weeks GA as a homogenous term group. One example is respiratory distress syndrome (RDS), which has as a relatively low absolute risk at late gestation yet demonstrates a gradient crossing term: 2.3% at 36 weeks, 1.2% at 37 weeks and 0.6% at 38 weeks [2]. Pulmonary immaturity however, is not the only complication of prematurity. Late preterm infants are more likely than term infants to need specialized care in the first few days [3-8] and experience minor maturity-related morbidity such as poor feeding, hypoglycemia, temperature instability and apnea [4,9-11]. More research is needed to examine whether every additional week of gestational age (GA) is associated with an improvement in outcomes in these areas, similar to what is seen with respiratory morbidity. Investigating this gradient is important since mean gestational age at delivery continues to shift to the left, with higher numbers of late preterm and early term deliveries [1,12,13].

Socioeconomic status (SES) is also linked with birth outcomes; lower SES groups demonstrate higher neonatal morbidity and mortality, partially related to their higher rates of preterm delivery [3,12,14-17]. Many infants thus have two interrelated risk factors for morbidity, GA and SES. Understanding the interactions between these two factors is important if we are to develop strategies for decreasing preterm birth and reducing neonatal morbidity [13,18,19]. Small decreases in morbidity per infant when many infants are affected have large public health and resource impacts [2,20].

The objective of this study is to examine how the neonatal morbidities related to prematurity persist into early term gestation and whether there is an independent effect of SES in a population based dataset.

## Methods

A retrospective cohort study was undertaken using the Population Health Research Data Repository (Repository) at the Manitoba Centre for Health Policy (MCHP). The Repository contains a number of anonymized administrative datasets collected by Manitoba Health, that capture health service utilization on the entire population of Manitoba, Canada (population = 1.2 million). This study utilized data from hospital discharge abstracts, Vital Statistics, and a population registry as well as public access Canada census files and welfare data from Family Services. The study cohort included all infants born at 34–41 completed weeks gestation during the fiscal years 2004/2005 to 2005/2006, who remained in Manitoba until their first birthday. As this was a study looking at prematurity-related complications, infants born at 42 weeks and above were not included. Birth records were linked to a maternal file for extraction of pregnancy related variables. All linkage was done using a unique identifier (scrambled personal health identification number). The validity and utility of this database has been previously documented [21-23]. Excluded from the cohort of 25 834 newborn records were 24 records that could not be matched with the population registry or maternal health records, 436 infants who moved before their first birthday and 62 which were missing birth weight, GA or income data, resulting in a final cohort of 25 312 infants. Income and GA distribution of those infants who moved did not differ significantly from those retained.

The first variable of interest was GA which was taken from the hospital record. GA was based on menstrual dates or ultrasound dating unless the clinical estimate at delivery differed in which case it was used. GA was entered as a categorical variable by completed week with 39–40 weeks GA as the reference group; preliminary models demonstrated no significant differences between 39- and 40-week infants in any model, thus they were grouped together. The second variable of interest was SES. Two different variables were used to assign infants to an SES group using maternal data. An individual level variable, receipt of provincial income assistance by the mother in the month of delivery, placed an infant in the lowest SES group. Additionally, average household income in area of mother’s residence from Canada Census data was assigned at the dissemination area level (approximately 400 persons) and grouped into population quintiles. In order to preserve sample size for the study of interactions between SES and GA the income quintiles were used to divide the population into 3 approximately equal sized groups. Infants in income quintile 1 were placed in the lowest group along with the income assistance recipients; quintiles 2 and 3 were placed in the middle group and quintiles 4 and 5 in the highest group. SES was analyzed using the highest group as the reference. Regression models were constructed including an interaction term for SES by GA, all other variables were additive. Other clinical variables were taken from the hospital abstract and included International Classification of Diseases (ICD) Version 10 codes as well as standard variables recorded for all newborn admissions.

The control variables were taken from the hospital abstract, and grouped where appropriate. They included: maternal diabetes, parity, maternal age group (under 19, 19–34 and over 34 years), infant gender, caesarean section delivery, induced delivery, multiple or singleton gestation, size for GA, congenital anomalies, rural residence, breastfeeding initiation and need for resuscitation at birth. As need for resuscitation at birth could be either a risk or an outcome, analysis with and without this variable was performed where appropriate. Reason for induction and type of caesarean delivery are not available in this data set. Congenital anomalies were those diagnosed over the first year of life and included only those expected to increase medical care. Large and small for GA infants were defined at the 90th and 10^th^ percentile for gender [24]. Need for resuscitation included those infants receiving positive pressure ventilation, chest compressions or drugs for resuscitation at delivery.

The primary outcome was infant morbidity during the birth hospitalization, analyzed overall as ‘any diagnosis.’ The following subgroups were also analyzed: respiratory distress syndrome (RDS), other complications of prematurity (apnea, hypoglycemia, temperature instability, poor feeding) and all other respiratory morbidity (transient tachypnea, pneumonia, persistent pulmonary hypertension, air leak syndromes, pulmonary hemorrhage, aspiration, respiratory arrest). Resource utilization was studied using two measures, admission to a Level II/III nursery (Special Care Nursery, NICU) and receipt of phototherapy.

Logistic regression models controlled for the effect of the previously described perinatal variables. As the primary outcome was ‘any neonatal morbidity’ power analysis using currently published morbidity rates of late preterm infants suggested an initial birth cohort size of approximately 28 000 was adequate to detect small differences (OR 1.2) with 80% power. Significance was set at p<0.05. All data handling and statistical analysis was done using SAS version 9.1 for Unix. Appropriate approvals were obtained from the custodians of all information sources in addition to the University of Manitoba Health Research Ethics Board and the Health Information Privacy Committee of Manitoba.

## Results

**Figure 1:**
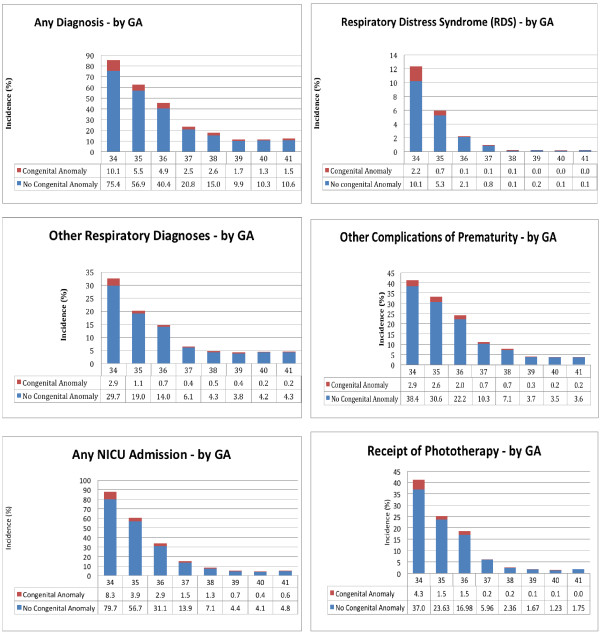
Unadjusted outcomes by gestational age at birth and presence of a congenital anomaly.

There were 25 312 infants in the birth cohort; for their characteristics see Table 1 (by GA) and Table 2 (by SES). Infants with congenital anomalies, 2.5% of the total infants, accounted for only a small proportion of morbidity as demonstrated in the unadjusted outcomes (Figure 1). In these graphs it is demonstrated that all outcomes decrease with increasing GA and this pattern continues past 37 weeks (Figure 1). For SES the patterns were mixed (Table 3) with the exception of the RDS outcome where there was a clear increase with increasing SES.

**Table 1:** Characteristics of study population by GA at birth and selected maternal and neonatal variables

**GA**	**total**	**34**	**35**	**36**	**37**	**38**	**39**	**40**	**41**
**n**	25312	276	457	801	1780	4189	6535	7218	4056
**Maternal Characteristics**									
maternal age group, years <19	5.1	5.8	3.5	4.2	4.9	4.4	5.4	5.1	5.5
>34	12.9	13.8	15.3	17.7	16.1	15.1	12.4	11.4	11.2
primiparous, %	38.1	44.2	39.6	36.6	35.7	32.5	35.1	39.4	46.8
caesarean section, %	20.8	35.9	33.7	29.0	24.9	30.1	21.2	13.9	17.2
induced delivery, %	26.8	26.8	35.2	35.0	33.7	34.8	22.1	16.4	39.1
multiple gestation, %	2.2	23.6	21.9	11.7	8.0	2.7	0.3	0.2	0.0
maternal diabetes, %	4.8	7.6	12.0	17.2	10.1	8.5	3.8	2.4	0.8
low SES, %	32.4	35.9	37.2	39.3	36.8	33.6	31.8	31.2	30.4
breastfed, %	81.6	68.8	69.4	73.9	79.2	80.5	81.3	83.2	85.3
rural, %	44.2	34.8	42.2	41.3	39.6	43.3	42.9	47.8	44.3
**Newborn Characteristics**									
male, %	51.2	58.0	55.4	54.2	51.7	53.0	49.6	50.1	51.9
mean BW, grams (SD)		2377 (460)	2640 (534)	2936 (530)	3143 (480)	3358 (479)	3496 (459)	3656 (463)	3774 (469)
BW range, grams		1184-3902	1216-4599	1420-5160	1172-5305	1610-6440	1703-6457	1349-5589	1438-5795
Large for GA, %	14.9	12.7	15.3	16.7	15.4	15.6	14.1	15.0	14.9
Small for GA, %	7.5	7.3	8.5	8.9	7.5	7.3	8.0	7.0	7.2
resuscitation at birth, %	7.6	31.2	20.1	12.2	9.2	7.3	5.5	6.7	7.9
median LOS, days (range)	2 (1-168)	13 (1-90)	6 (1-66)	3 (1-71)	2 (1-103)	2 (1-168)	2 (1-93)	2 (1-55)	2 (1-42)
major congenital anomaly, %	2.5	10.1	5.7	5.6	3.2	3.1	2.1	1.7	2.1

Legend: *SES* = socioeconomic status, *BW* = birthweight, *CS* = caesarean section, *LOS* = length of stay.

**Table 2:** Characteristics of study population by SES and selected maternal and newborn characteristics

**SES group**	**Lowest**	**Middle**	**Highest**
n	8203	8854	8255
**Maternal characteristics**			
maternal age group, years			
<19	10.3	3.3	1.7
>34	8.1	12.9	17.5
primiparous, %	33.3	40.2	40.6
CS, %	17.5	21.9	23.0
maternal diabetes, %	7.2	4.2	3.0
induced delivery, %	25.5	27.9	26.9
multiple gestation, %	1.7	2.0	2.8
rural, %	45.8	44.5	42.3
breastfed, %	69.1	84.8	90.6
rural, %	45.8	44.5	42.3
male, %	51.3	51.4	51.0
mean BW, grams (SD)	3482 (554)	3505 (534)	3492 (526)
BW range, grams	1216– 6208	1172– 6457	1331– 5746
Large for GA, %	16.0	15.0	13.8
Small for GA, %	8.0	7.4	7.0
resuscitation at birth, %	8.0	7.6	7.0
median LOS, days (range)	2 (1–103)	2 (1–168)	2 (1–51)
premature, %	7.1	5.4	5.7
major congenital anomaly, %	2.8	2.6	2.1

*BW* = birthweight, *CS* = caesarean section, *SES* = socioeconomic status.

**Table 3:** Unadjusted outcomes by SES group

**SES group**	**Lowest**	**Middle**	**Highest**
Any diagnosis, %	17.7	17.2	13.9
RDS, %	0.4	0.5	0.7
Other complications of prematurity, %	7.6	6.7	5.5
Other respiratory, %	5.5	6.2	4.8
NICU admission, %	10.1	9.4	7.7
Phototherapy, %	3.9	3.2	2.6

Models were constructed to test for an interaction between GA and SES but none was demonstrated in any of the outcomes and the interaction term was dropped. Models with removal of the ‘need for resuscitation’ variable did not change the direction or significance of the reported results. The adjusted outcomes by GA and SES can be found in Table 4. Further data for the variables used in the adjusted outcomes can be seen in Additional file 1. For respiratory outcomes a higher odds ratio was seen at 34–37 weeks GA and mixed results were seen for low SES infants; for RDS there was a trend towards a decreased odds ratio, and in other respiratory outcomes this trend was significant but in two directions. For associations overall, for non respiratory morbidities and for NICU admission the odds ratio remained higher at 34–38 weeks GA and the odds ratio decreased as SES increased.

**Table 4:** Odds ratios (95% CI) for logistic regressions modeling associations between GA and SES for primary and secondary outcome groups

**Morbidity group**	**Any diagnosis (n = 4120)**		**RDS (n = 135)**		**Other complications of prematurity (n = 1167)**		**Other respiratory (n = 1392)**		**NICU admission (n = 2299)**		**Phototherapy (n = 819)**	
**Independent variables**	**OR**	**95% CI**	**OR**	**95% CI**	**OR**	**95% CI**	**OR**	**95% CI**	**OR**	**95% CI**	**OR**	**95% CI**
**Gestational age (weeks)**												
34 vs 39/40	**39.8**	27.8–57.1	**32.6**	17.8–60.0	**13.4**	10.1–17.8	**5.8**	4.3–7.9	**126.4**	85.2–187.5	**40.4**	29.7–55.0
35 vs 39/40	**11.1**	8.9–13.8	**19.6**	10.7–36.2	**8.9**	7.7–11.3	**3.5**	2.7–4.6	**25.9**	20.6–32.7	**20.2**	12.3–26.6
36 vs 39/40	**5.0**	4.3–6.0	**9.2**	4.8–17.7	**4.8**	3.9–5.9	**2.9**	2.3–3.6	**7.4**	6.2–9.0	**11.8**	9.2–15.1
37 vs 39/40	**1.9**	1.7–2.2	**4.5**	2.4–8.6	**2.1**	1.8–2.6	**1.3**	1.0–1.6	**2.8**	2.4–3.3	**3.9**	3.1–5.0
38 vs 39/40	**1.3**	1.2–1.5	1.0	0.5–2.2	**1.5**	1.3–1.8	0.9	0.8–1.1	**1.4**	1.2–1.6	**1.5**	1.2–2.0
41 vs 39/40	**1.0**	0.9–1.1	0.9	0.4–2.1	1.0	0.8–1.2	1.0	0.8–1.1	1.0	0.9–1.2	1.1	0.9–1.5
**SES group**												
Low vs high	**1.2**	1.1–1.4	0.7	0.5–1.0	**1.3**	1.1–1.4	1.1	0.97–1.3	**1.2**	1.1–1.4	**1.5**	1.2–1.8
Middle vs high	**1.3**	1.2–1.4			**1.2**	1.1–1.4	**1.3**	1.2–1.5	**1.3**	1.2–1.5	**1.3**	1.0–1.5

*low vs all other due to sample size. Models adjusted for: multiple gestation, maternal diabetes, infant gender, size for GA, congenital anomalies, need for resuscitation at birth, induced delivery, caesarean section delivery, primiparous, maternal age group, rural residence and breastfeeding initiation.

**Figure 2:**
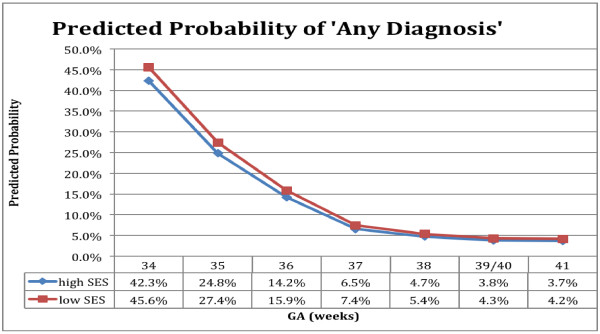
**Predicted probability of any diagnosis during birth hospitalization, for reference infant, by GA and SES.** Legend: Reference infant: singleton female, appropriate size for GA, no congenital anomaly, no resuscitation at birth, no maternal diabetes, born via non-induced vaginal delivery, multiparous mother from the highest income and middle age group, not rural, with breastfeeding initiation.

A graph predicting ‘any diagnosis’ (Figure 2) was generated from the regression model for descriptive purposes. The reference infant was a singleton female of appropriate size for GA with no congenital anomaly who required no resuscitation at birth, was born via non-induced vaginal delivery to a multiparous mother from the highest income and middle age group without diabetes, residing in an urban center who had initiated breastfeeding. These characteristics were chosen as they did not confer increased risk of morbidity within the model.

## Discussion

This study evaluated two primary determinants of neonatal morbidity, GA and SES, after adjusting for multiple maternal and fetal confounders in a large population based sample. It included infants cared for in many different areas including rural and urban, community and tertiary care centers, normal newborn and intensive care nurseries. The lack of individual level markers of SES was overcome using area level data, which have previously been demonstrated as appropriate for assessing the association between SES and health outcomes [22,25]. SES and GA were found to be independently associated with neonatal morbidity; that is, the effect of SES was the same regardless of the GA of the infant. The often detrimental association of low SES with outcomes is as important for term as for preterm infants; if the association with SES is causal it suggests that interventions to improve SES will be helpful across all gestational age groups.

The potential weakness of the study is inaccuracy of coding in administrative data. The consistent gradient relationship in outcomes suggests that small errors in GA estimation do not distort the findings. The population registry in this dataset has been demonstrated to provide excellent ascertainment of cohorts [23] and few records were lost for non linkage. Hospital discharge abstracts have demonstrated agreement with re-abstraction studies for capture of serious comorbidity and primary reason for admission with less ability to capture minor comorbidity, which is often under-captured [22]. Two techniques to overcome this are grouping of diagnoses as well as larger sample sizes. In addition, despite lower capture rates the predictive value of the data was similar in these studies to that from a more comprehensive clinical dataset. These data are most useful in identifying overall patterns in healthcare usage and diagnosis, with detailed studies requiring more clinical precision. Our findings are consistent in their direction and agree with other published studies using non-administrative datasets.

Our study confirms previous reports of higher short-term respiratory morbidity in late preterm (3.2–40%) compared to term (1–5%) deliveries [10,11,26-29]. Further to this we have demonstrated that these groupings do not tell the whole story, as increased risk is seen beyond the preterm period, at 37 weeks, after adjusting for previously reported risk factors such as induction of labour and caesarean delivery [30,31]. Increased respiratory risks in non elective deliveries at 37–38 weeks have previously been demonstrated [32,33]. We have demonstrated increased risks in a combined population of elective and non elective deliveries after control for induction regardless of indication, as this information is not available. It is important to acknowledge that some of the recent increase in late preterm and early term birth is due to induction for fetal or maternal wellbeing and has resulted in decreased stillbirth rates, especially in multiple gestation pregnancies and this is why we have controlled for these factors.

In addition to higher respiratory morbidity in early term infants we also demonstrate higher non respiratory morbidity. Smaller cohort studies have previously demonstrated an increase in composite morbidity [11,16] in early term infants, as have studies of elective deliveries only [31,34]. Increased non respiratory morbidity in early term infants has been demonstrated in a cohort of infants with mature fetal lung indices [35]. Our study has a larger, population based sample of all deliveries with greater detail of the outcomes under study and confirms these findings after adjustment for both medical and social risk factors. Our findings agree with a population based study of over 150 000 infants by Gouyon et al. [36], that demonstrated increased risk for neurologic morbidity in late preterm infants which persisted in 37 week infants but did not report on other non respiratory morbidity.

Our study demonstrates a small but consistent association of low SES with morbidity after controlling for GA. As our low SES group accounts for approximately a third of the population these small increases translate into large numbers of infants at increased risk at the population level, over 4000 infants per year in Manitoba. Studies reporting the associations between neonatal morbidity and SES are few and mixed, depending on markers used and outcomes studied [11,18]. Less clear is the association between SES and respiratory morbidity in our study where we demonstrated a trend towards decreased prevalence of RDS in low SES infants and an unclear pattern for the effect of SES on other respiratory outcomes, possibly due to small numbers. These trends were seen after adjustment for other factors that vary with SES such as maternal diabetes and mode of delivery. There is emerging research that maternal stress impacts fetal outcomes via the corticosteroid pathway [37]. Women from lower SES groups have higher baseline stress [17] which could lead to fetal lung maturity at earlier gestations, similar to the effect seen in growth restricted fetuses [38].

These data have demonstrated in all outcome groups that the morbidities classically associated with prematurity persist well into term gestation and underline the importance of week by week analysis; a term control group containing a high percentage of early term infants could have morbidity approaching that of a late preterm group. As GA increases so does the number of infants born at that GA (Table 1), thus small percentages translate into large numbers of infants at risk and high resource usage. While the severity of these morbidities is often not high they do lead to medical interventions and parental anxiety. Separation of mother–infant pairs can have long lasting effects [39] especially with respect to initiation of breastfeeding, already at risk due to late preterm and one could postulate early term birth.

## Conclusions

The persistence of elevated morbidity into early term gestation has practical implications for practitioners and policy makers with the first step being recognition. The gradient in morbidity underlines the importance of risk versus benefit considerations when semi-elective delivery is planned before 39 weeks; this must include not only consideration of respiratory but other morbidities as well as their impact on the healthcare system, particularly bed and personnel availability. Hospital policy should clearly discriminate between elective and semi-elective deliveries, and lung maturity should not be the only factor in determining readiness for induction. Educational and research initiatives such as those of AWHONN [40] and The National Institute of Child Health and Human Development [41] aimed at late preterm infants should be extended to include early term infants to improve our knowledge and care of these infants. Families should be educated about the gradient in maturity, especially when involved in discussions about timing of delivery. Absence of traditional risk factors for morbidity such as maternal diabetes, induction and caesarean delivery is not synonymous with absence of risk. Recognition of SES as an independent risk factor is important and an income based measure as used in this study is but one facet of the social determinants of health. Pregnancy is often a window of opportunity for health care providers to address lifestyle factors such as smoking, substance use and control of diabetes and to build ongoing relationships with families to improve continuity of care and maternal health [42]. Physicians and health care providers should ensure that their patients are aware of programs available to them to provide financial, educational and social support and ultimately improve health outcomes.

## Competing interests

None declared by any authors.

## Authors’ contributions

CAR: Conception and design of the study, acquisition, analysis and interpretation of data, drafting and revising the article, final approval of the version to be published. NP, EH-R, MD: Design of the study, interpretation of data, revising the article, final approval of the version to be published. All authors read and approved the final manuscript.

## Supplementary Material

Additional file 1 Odds ratios with 95% confidence intervals for variables used in the logistic regression models to generate adjusted outcomes by GA and SES. Click here for file
