# Supplementary material for: ‘The influence of gestational age and socioeconomic status on neonatal outcomes in late preterm and early term gestation: a population based study’
Source: BMC Pregnancy Childbirth. 2012 Jun 29;12:62. doi: 10.1186/1471-2393-12-62 (PMC3464782; doi:10.1186/1471-2393-12-62)
Supplement: Additional file 1 — Odds ratios with 95% confidence intervals for variables used in the logistic regression models to generate adjusted outcomes by GA and SES. [file 1471-2393-12-62-S1.pdf]

Morbidity Group	Any Diagnosis (n=4120)		RDS (n=135)		Other complications of prematurity (n=1167)	
**Control Variables**	**OR**	**95% CI**	**OR**	95% CI	**OR**	95% CI
**Maternal diabetes**	**4.1**	3.5-4.7	**0.4**	0.2-1.0	**8.1**	6.9-9.5
**primiparity**	**1.6**	1.5-1.8	0.9	0.6-1.4	**1.7**	1.5-2.0
**maternal age group (yrs)**	p=0.10		p=0.86			
<19 vs 19-34	0.9	0.7-1.0	0.9	0.4-2.3	**0.7**	0.5-0.9
>34 vs 19-34	1.1	0.9-1.2	1.1	0.7-1.9	1.1	0.9-1.3
**induced delivery**	**1.4**	1.3-1.5	1.3	0.9-2.0	**1.4**	1.2-1.6
**male infant**	**1.2**	1.1-1.3	**2.0**	1.4-3.0	1.1	0.9-1.2
**caesarean section**	**1.5**	1.4-1.7	**1.9**	1.2-2.9	**1.4**	1.3-1.6
**multiple gestation**	0.9	0.8-1.2	1.4	0.9-2.4	1.2	0.9-1.5
**SGA**	**1.6**	1.4-1.8	0.6	0.3-1.2	**1.8**	1.5-2.2
**LGA**	**1.5**	1.4-1.7	0.9	0.5-1.6	**1.5**	1.3-1.7
**any congenital anomaly**	**28.8**	23.2-35.7	**3.0**	1.7-5.2	**2.6**	2.1-3.3
**rural residence**	1.0	0.9-1.1	NA		**0.8**	0.7-0.9
**any breastfeeding**	1.0	0.9-1.1	NA		1.0	0.8-1.1
**need for resucitation**	**3.7**	3.3-4.1	**2.0**	1.6-2.4	**1.7**	1.4-2.0

Morbidity Group	Other respiratory (n=1392)		SCU (n=2299)		phototherapy (n=819)	
**Control Variables**	**OR**	95% CI	**OR**	95% CI	**OR**	95% CI
**Maternal diabetes**	1.2	0.9-1.5	**2.8**	2.4-3.4	**2.3**	1.8-2.9
**primiparity**	1.2	1.0-1.3	**1.3**	1.2-1.4	**1.9**	1.6-2.3
**maternal age group (yrs)**	p=0.86		p=0.32		p=0.16	
<19 vs 19-34	0.8	0.6-1.0	0.9	0.7-1.1	1.1	0.8-1.5
>34 vs 19-34	0.9	0.8-1.1	0.9	0.8-1.1	1.2	0.99-1.5
**induced delivery**	1.1	0.99-1.3	**1.5**	1.3-1.6	**1.3**	1.1-1.5
**male infant**	**1.4**	1.3-1.6	**1.3**	1.2-1.4	**1.2**	1.0-1.4
**caesarean section**	**1.8**	1.6-2.1	**1.5**	1.4-1.7	1.0	0.8-1.2
**multiple gestation**	1.0	0.7-1.3	**1.4**	1.1-1.8	**0.6**	0.4-0.8
**SGA**	0.8	0.7-1.1	**2.7**	2.3-3.1	**2.3**	1.8-2.9
**LGA**	1.1	0.9-1.3	**1.3**	1.1-1.5	**1.6**	1.3-2.0
**any congenital anomaly**	**2.2**	1.7-2.8	**6.4**	5.2-7.8	**1.7**	1.2-2.3
**rural residence**	**0.9**	0.8-0.99	**0.5**	0.4-0.5	1.0	0.9-1.2
**any breastfeeding**	0.9	0.8-1.0	**0.7**	0.7-0.8	**1.3**	1.0-1.6
**need for resucitation**	**6.6**	5.8-7.5	**4.6**	4.1-5.3	**1.5**	1.2-1.8
